# S3 and Recovery and Resilience Funds: A Case Study Built on the Experience of 10 Spanish Regions

**DOI:** 10.3389/frma.2021.801370

**Published:** 2022-01-06

**Authors:** Cecilia Gañán de Molina, José Emilio Guerrero Ginel, Carmen Sillero Illanes

**Affiliations:** Agricultural, Food, Forestry Engineering and Sustainable Rural Development, University of Córdoba, Córdoba, Spain

**Keywords:** smart specialisation, regions, recovery, multi-level governance, innovation

## Abstract

The EU's response to the COVID-19 crisis, namely the approval of the Next Generation package, provides an opportunity to explore to what extent the existing Smart Specialisation regional strategies and related ecosystems have been taken into account in the highly relevant territorial context in which the national Recovery Plans have been designed. According to our results the potential of the Smart Specialisation approach (S3) in relation with its place-based strategic prioritisation may have been overlooked in the process. The research is based on a desk review of relevant documents and recent literature in this field; followed by semi-structured interviews with regional planners and practitioners from 10 Spanish regions (autonomous communities); complemented, in a second phase, by the organisation of a focus group to validate the initial results. During our research we identified the main contributions that the Smart Specialisation approach has so far made to the regions (mainly in terms of participative governance and creation of regional ecosystems); and the unanimous perception shared by all the practitioners interviewed that the S3 approach has led to a change of vision in public intervention. However, all of the interviewed regions have confirmed that the drafting of the national recovery and resilience plan lacked an ex-ante alignment with the regional S3 strategies, and failed to consider the existing regional S3 ecosystems. The separation of the recovery logic (based on the operation of public consultations at national level to identify strategic projects) from the S3 logic (based on a strategic prioritisation exercise conducted by each regional ecosystem) confirms that an opportunity may have been missed in the recovery planning process to consolidate the multi-actor, multilevel and place-based S3 approach. Although there is a certain degree of disappointment among regional practitioners as a result of this misalignment, the majority of them believe in the possibility of an ex-post alignment between the two processes, that can protect existing regional shared visions. However, without clear recognition of the S3 ecosystems and the S3 managing bodies, the significant role that Smart Specialisation could play in the recovery process may be at risk.

## Introduction

Smart Specialisation (S3) is a policy approach consisting of an integrated, place-based economic transformation agenda focusing policy support and investments on priorities linked to the strengths and competitive advantages of each region. Under this approach, priority domains are identified using the “entrepreneurial discovery process” (EDP) which is what distinguishes S3 from former strategies (OECD, 2013), and it has been described as “an effective tool to maximise the innovation, entrepreneurial and growth potential of every territory” (Rodríguez-Pose, 2017). This strategy became an ex-ante condition for Member States and regions to have access to European funds under the 2014–2020 Multiannual Financial Framework (MFF). This is why the Research and Innovation strategies for Smart Specialisation (RIS3) have been guiding research and innovation investments of over EUR 40 billion from the European Regional Development Funds (ERDF)—over EUR 65 billion if national co-financing is included—between 2014 and 2020 (Hegyi et al., 2021). In the European Cohesion Framework 2021–2027^1^, the conditionality role of the Smart Specialisation Strategies has been reinforced. From being considered as an “ex ante condition” for regional programming under the European Development Fund (ERDF) Objective 1, in the current framework, the “Good governance of national or regional smart specialisation strategy” has become a thematic “enabling condition” for a wider Policy Objective 1, “Smarter Europe by promoting innovative and smart economic transformation” applicable not just to the ERDF, but also to the European Social Fund (ESF) and Cohesion Fund.

Inter-government coordination emerged in the EU regions thanks to the S3 approach (Guzzo and Gianelle, 2021) and has received increasing attention since its implementation. As a result of that, new rules and arrangements have been agreed; and a new set of capacities and a heightened awareness have been created at regional level. However, the implementation of the S3 approach creates challenges in all types of regions (Trippl et al., 2020) and calls in particular for new forms of experimental governance at national and subnational levels (Gianelle et al., 2020), especially in cases where regional and national governments have already been recognised as managing authorities and see multilevel governance as a threat to their centrality in the strategy (Larrea et al., 2019). Some authors actually consider that this policy approach “contains a number of hidden assumptions about the political dynamics of regional innovation policy” some of which they term “heroic” because they are so challenging, particularly for public authorities in the regions needing the most help (Marques and Morgan, 2018). Especially challenging is the concept of “multilevel governance” that is closely linked to the S3 approach. This concept was central to the proposals made by the European Commission early in the previous decade in terms of the suitability of establishing “a permanent dialogue between various levels of government” and of “extending cooperative approaches to national, local and regional authorities to social agents, stakeholders and civil society” (European Commission, 2010). In practise, this “permanent dialogue” has proven to be very demanding, especially when it occurs between different levels of government that hold different mandates and responsibilities (Cohen, 2019). This is the case in countries like Spain, where the high level of territorial autonomy of the regions (autonomous communities) allows them a high degree of policy-making autonomy while remaining subject to national government decisions on strategic national programmes.

Accordingly, the purpose of this paper is to assess Spanish S3 regional practitioners' experience of the implementation of Smart Specialisation strategies (RIS3) between 2014 and 2020 and the extent to which the Smart Specialisation approach and related ecosystems have been taken into account in the process of drafting the national Recovery and Resilience Plan.

National Recovery Plans have been prepared by the Member States in response to the adoption, in July 2020, of the recovery-oriented plan “Next Generation EU for the years 2021–2024” (European Commission, 2020), the main mechanism of which is the Recovery and Resilience Mechanism which has committed a budget of EUR 750 billion (EUR 390 billion of which will be awarded in the form of non-refundable grants). Marques Santos (2021) considers that in a singular context of recovery, short-term decisions must be aligned with medium/long-term targets and, accordingly, that the process of entrepreneurial discovery under Smart Specialisation could have helped to identify such a match. In that connexion, the integration of regional initiatives into the core of national recovery and resilience strategies should have emerged from the outset as a decisive factor to be taken into account in the drafting of recovery plans. Along the same lines, recent studies by the Organisation for Economic Co-operation and Development (OECD, 2021) highlight the importance of introducing, activating or reorienting existing multi-level coordination bodies that bring together national and subnational government representatives to minimise the risk of a fragmented crisis response; and many voices advocate: (1) early involvement of subnational governments in national investment recovery strategies to ensure that allocation criteria are guided by strategic regional priorities (OECD, 2019; Corpakis et al., 2020; Magro et al., 2020); (2) alignment with the EU green and digital transitions (Magro et al., 2020); and (3) consideration of strategic planning exercises already in place in in the regions, such as the one offered by the S3 (Wilson et al., 2020). Furthermore, stakeholder involvement (and regional S3 ecosystems should be considered as key stakeholders in the national recovery framework) should be seen as a pre-condition for a successful continuous entrepreneurial discovery process (Marinelli and Perianez-Forte, 2017).

Despite all these considerations, the preparation of the national recovery plans has prompted a debate on the degree of maturity of multilevel governance in the Member States, since regions and regional S3 ecosystems have not been invited to take part in their preparation, as the Committee of the Regions has warned on several occasions.^2^

In order to study the extent to which the Spanish national recovery plan has taken account of the existing regional S3 strategies, priorities and ecosystems, our research is framed on the stakeholder analysis methodology, which has gained increasing recognition by providing a tool for analysing how far stakeholders and their characteristics (mainly interests, position and power—Gilson et al., 2012), can influence decision-making (Slabá et al., 2019). Specifically, we based our research on the perceptions of Spanish regional S3 practitioners, as highly relevant actors both in the regional priority-setting process developed under the S3 approach; and in the framework of the strategic parameters for the recovery process conceived at national level.

As our study shows, for S3 to achieve its full potential, there is a need for an upgrade in the quality of multilevel governance to consolidate the consideration of regional priorities established under this approach; a strengthening of capacities to perform S3-associated policy functions (according to Guzzo and Gianelle, 2021; Hegyi et al., 2021); and greater recognition of the contributions that S3 regional ecosystems can make at the broad European policy-making level. This would help to secure what Foray (2015) and Radosevic (2017) term “pockets of administrative excellence” which will be needed to effectively implement the transformational potential of S3 in the framework of the European recovery.

## Materials and Methods

To achieve our goal, we began with a desk review of the literature regarding S3, paying special attention to the aspects related to governance, inter-government coordination, innovative approaches in public intervention and S3 regional ecosystems (Marinelli and Perianez-Forte, 2017; Radosevic, 2017; Marques and Morgan, 2018; Larrea et al., 2019; Gianelle et al., 2020; Magro et al., 2020; Trippl et al., 2020; Guzzo and Gianelle, 2021; Hegyi et al., 2021). We supplemented this review with the literature concerning S3 in Spain which has focused on a number of aspects such as the early challenges of RIS3 in Spain (del Castillo et al., 2015); the validity of the RIS3 approach regarding disadvantaged regions (Madeira et al., 2021); the role of Universities (Pérez et al., 2017); and vocational education and training and RIS3 (Moso-Díez, 2020). Very few references were found in the recent literature, however, to multilevel governance between regional S3 strategies and the Resilience and Recovery planning process.

The desk review was followed by the identification of our main target group, and for that purpose we considered the contributions made by the stakeholder analysis methodology, a useful tool for generating knowledge about the relevant actors in a specific process or planning exercise so as to understand their behaviour, intentions, interrelations, agendas, interests, etc. and assess future policy directions (Brugha et al., 2000). A complex process such as the drafting of a national recovery plan involves many stakeholders (companies, governments, policy-makers, academia, etc). In our study we are focusing on the perceptions and experience of one specific group of stakeholders only, but a highly relevant one: the regional S3 practitioners who, as our research confirms, may have been marginalised or ignored (and with them the whole S3 regional ecosystem that they embody) during that process.

For our purpose, we first conducted a set of semi-structured individual interviews followed by a focus group with all the participants. A recent review on stakeholder analysis confirms that 63% of the studies analysed are based on interviews (Bendtsen et al., 2021), while the focus group is regarded as an efficient research approach for collecting knowledge from multiple participants, and as an appropriate tool for democratising the research process (Ledger et al., 2019).

The interviewees were selected, as explained above, on the basis of their role as stakeholders handling the planning of S3 strategies at regional level. In May 2021 we conducted the semi-structured individual interviews with S3 regional practitioners of 10 Spanish regions (Autonomous Communities—Regions, NUTS2): Andalucía, Aragón, Asturias, Castilla-León, Cataluña, Extremadura, Galicia, Murcia, Navarra and Valencia. The interviews were confidential, gender-balanced (7 women and 6 men), and were conducted by the three authors *via* videoconference between 6 and 25 May 2021. The conversations lasted 45–60 mins and the relevant documentation was provided in advance.

In order to define the scope of the questions to be asked in the interviews, the team of authors prepared an initial set of ideas which were framed (a) in Smart Specialisation theory and its practical application in European regional policy since 2014 (this policy attributed a conditionality role to S3 that has now been reinforced in the current European Cohesion Framework 2021–2027); and (b) in the literature in the field, which has made an in-depth study of the importance of multilevel governance for a successful implementation of the S3 approach; the key role of S3 regional units in implementing innovative S3 mechanisms in public intervention; and the strategic contributions that regional S3 ecosystems can make to ensure multi-actor and place-based priority-setting in the autonomous communities.

The initial ideas for the interviews were reformulated after the two initial pilot interviews which helped to give final shape to the questions: (1) What are S3's main contributions in the region? (2) What are the main difficulties in implementing the S3 approach? (3) What is the relationship between S3 processes and the Recovery Mechanism-related process? (4) Are the two processes aligned?

In the second phase, on 15 October 2021, seven months after the interviews were held, a focus group was organised by the three authors, in which 7 of the 10 regions participated, and they were asked to validate and discuss the preliminary results of the study. The focus group methodology has its origins in a group interviewing approach which was first described by Merton and Fiske (1956). It can be defined as “a type of group discussion about a topic under the guidance of a trained group moderator” (Stewart, 2018) that has the potential to allow a fluent articulation of implicit opinions and preferences, and may enable triangulation based on multiple perspectives and information (Ignjatović, 2020). Unlike in interviews, the role of the research team is peripheral (O. Nyumba et al., 2018) and requires a skilled facilitator and an assistant documenting the general content of the discussion (Kitzinger, 1994). The authors of this paper took on these roles as a team during the focus group organised with the stakeholders interviewed.

Focus group discussion usually consists of four major steps (Morgan and Krueger, 1998) comprising (1) research design; (2) data collection; (3) analysis; and (4) reporting of results. For the organisation of our focus group, we followed this scheme, basing the initial research design on the drafting of a starting document, the main purpose of which was to pre-assess the degree of alignment between the S3 regional process and the national recovery process. This document (which was circulated to the focus group beforehand) was drafted once the bilateral interviews had been held and included an initial set of questions to be discussed at the focus group level.

The focus group was organised on a confidential basis, enabling a non-threatening environment to be created, which is a key feature of this approach (Morgan, 1992). The data collection and the analysis of results enabled the authors to confirm the pre-identified patterns already detected during the first stage of semi-structured interviews, and to complement them with additional insights arising from group interaction.

Although the literature recommends reconvening a group for subsequent meetings, it has been also acknowledged that this can be difficult owing to changes in both personnel and circumstances (Bloor et al., 2001). As the actors taking part in our research had little time to attend subsequent focus group discussions, we conducted a single focus group meeting in which 8 out of the 10 actors participated (it is generally accepted that between six and eight participants are sufficient for a focus group to be effective—Krueger, 2000).

**Figure 1:**
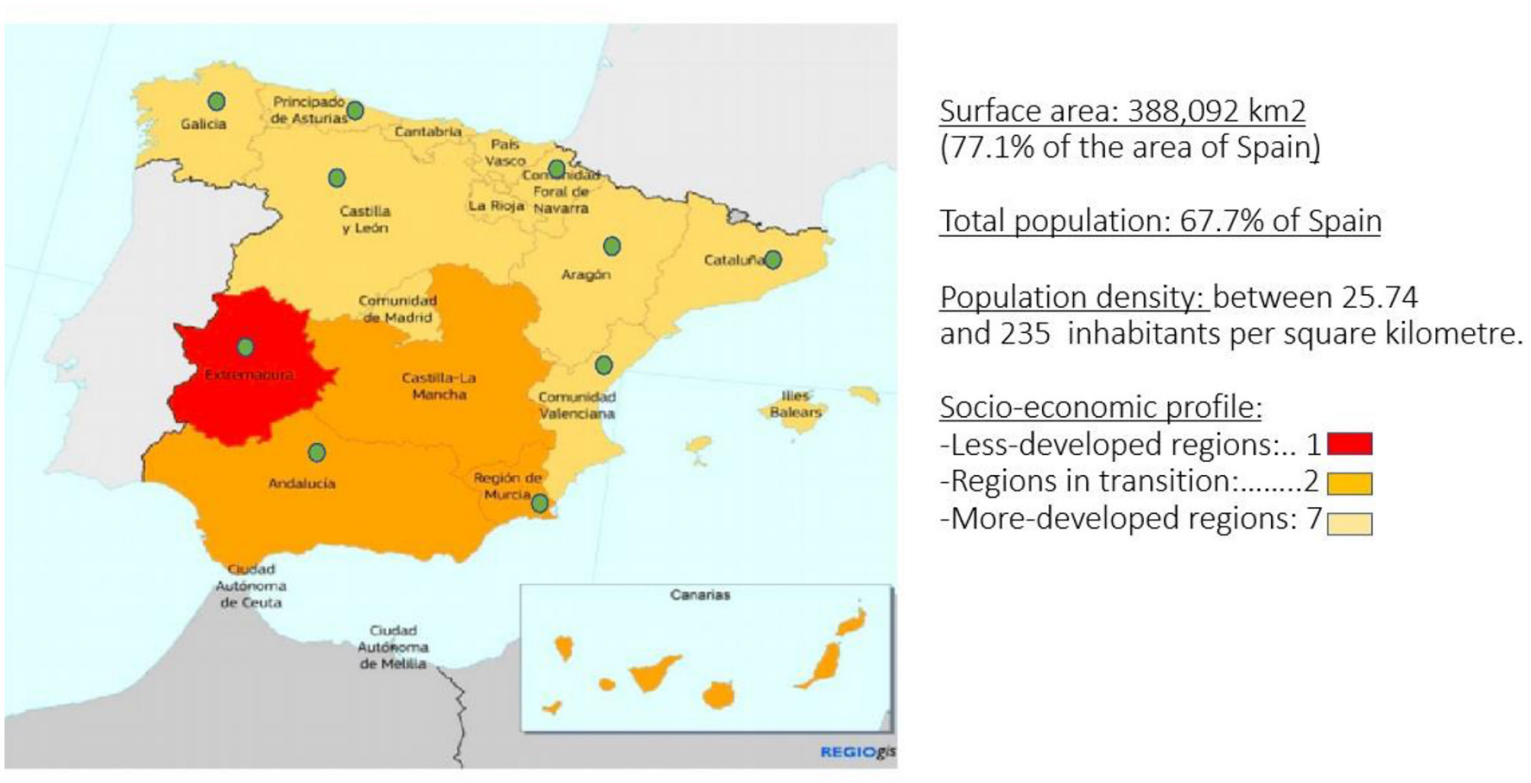
Map of Spanish regions participating in the case study. Source: Prepared by the authors from official data.

Finally, we would like to highlight that the selected stakeholders are highly representative since they cover almost the entire territory of Spain, which is one of the Member States that is to receive the largest amount of recovery funds over the coming years. In the figure below the 10 participating regions are identified by a green dot on the map (Figure 1).

## Results and Discussion

### Main Contribution of S3 in the Period 2014–2020

The first question addressed to the regional S3 practitioners was related to the main benefits that S3 has delivered to each region in its first period of application, 2014–2020. The majority of the interviewees identified the Entrepreneurial Discovery process together with the new planning methodology and its impact in a participatory governance as the main contribution of S3. The regions are now more aware of the importance of complementary actions, joint initiatives and multilevel governance; and have incorporated assessment routines on processes and impacts. These findings are consistent with Szerb et al. (2020) who argue that the benefits of S3 tend to be multi-dimensional rather than purely technological and research-related, also involving institutional and governance dimensions.

The regional S3 practitioners are also unanimous in considering that the creation of ecosystems has been key in the generation of innovative communities; and that S3 has allowed shared visions to emerge in a new context of co-responsibility, in which the existence of key relevant actors has played a strategic role, in accordance with, Hegyi et al. (2021) who consider that the existence of leaders can help new narratives to develop and thrive. The interviewees also mention as a positive contribution the option for private regional firms to open up to internationalisation within the scope of interregional S3 partnerships, although a much more robust integration for small and medium-sized enterprises (SMEs) is needed.

Finally, the interviewees agree that S3 is a very important driver for innovation in public policy, especially in relation to new and innovative governance approaches, as illustrated by an example shared by one of the regional actors taking part in the study: the http://catalunya2020.gencat.cat/web/.content/00_catalunya2020/Documents/estrategies/fitxers/agendes-compartides.pdf Shared agendas for sustainability and social change, which aim, through a model of participatory governance, to coordinate collective action to face common challenges in the region. Inspired by S3, these agendas are based on intersectoral cooperation and knowledge-sharing between public administrations, academia, companies and civil society which, specifically in the current recovery context, can be viewed as strategic tools at regional level (Generalitat de Catalunya., 2020).

However, the interviewees agreed that capacity needs to be built in the public sector in areas such as negotiation skills for ensuring successful multi-level governance and joint public-private implementation [in accordance with what Marques and Morgan (2018) consider “heroic assumptions” of S3]. Indeed, one of the regional practitioners interviewed, after mentioning the green and digital transition on which the EU has embarked, wondered when the public administration transition was going to happen.

**Figure 2:**
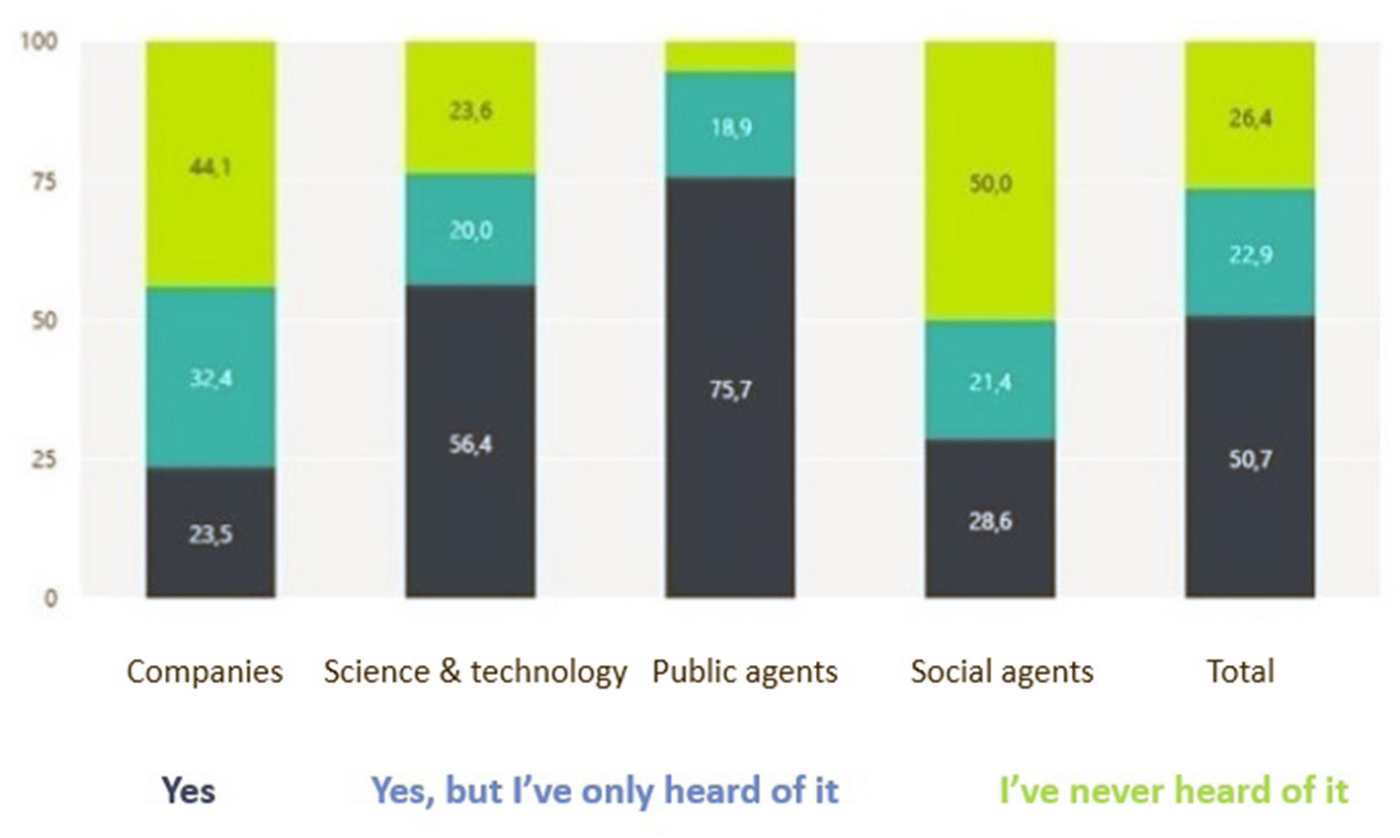
Extent of knowledge of RIS3 in the Valencia region (Spanish autonomous community), 2020. Source: “Analysis of the Valencian economy and the Valencian innovation system. Diagnosis in the current context” (Reig et al., 2020).

Another relevant barrier identified by the regional practitioners was related to communication, as reported by Larosse et al. (2020) who consider that the implementation of Smart Specialisation as a new policy concept has yet to take off, and needs to be better understood. By way of example, one of the participants shared with us the recent results of an analysis of its regional innovation system which reveals little knowledge of the concept of Smart Specialisation among its regional stakeholders (Figure 2).

### The Lack of Ex-ante Coherence Between S3 and the Recovery Funds

In September 2020, the European Commission presented strategic guidelines to the Member States in order to orient national recovery plans, to be implemented through the funding of Strategic Projects for Economic Recovery and Transformation. In the case of Spain, these projects were defined as strategic and with a high capacity to drive economic growth, employment and the competitiveness of the Spanish economy, with a major component of public-private cooperation across the various administrations. In order to receive expressions of interest in this regard, the Spanish national administration launched 14 public consultations in the fields of industry, green and digital transition, the demographic challenge and combating depopulation.

As a result of this process, on 30 April 2021 the Spanish Government officially presented the final “Recovery, Transformation and Resilience Plan,” which was favourably received by the Commission and was finally approved on 16 June 2021. The Plan (costing an estimated total of EUR 69 528 050 000) is expected to mobilise private investment across several sectors, including sustainable and clean energy and transport, building renovation, the agri-food sector, fisheries, health and key digital technologies.

Although in the opinion of the national Government the National Recovery Plan has been carried out in a manner that is consistent with S3 planning, in our survey, the regional S3 practitioners unanimously reported a lack of coordination between the two planning processes. The S3 regional units were not invited to take part in the process, nor to assess the coherence of the recovery projects with the S3 regional priorities. Actually, only 3 out of 10 regions are currently in a position to ensure coherence between the S3 strategy and the recovery projects, but there is a reason for that. Two of them because of their small size (which has easily allowed key iconic recovery projects to be identified in the territory); and the third thanks to the responsibilities of the S3 unit, which is also tasked with managing European funds in the region.

The most relevant thoughts shared by the interviewees on this lack of coordination included the following: the recovery projects were not associated with any ex-ante alignment with S3; there has been a disconnect between the logic of recovery and of RIS3, based on prioritisation by a regional ecosystem; there has been a very low level of regional participation; the new generation of S3, S4, (with a greater emphasis on sustainability as a strategic driver of the new strategies) has been diluted as a regional priority.

Although there is a certain amount of understanding of the complexity and urgency of the context to explain the current situation and the speed of the process, the feeling of the regional S3 practitioners is that public consultations have not been well targeted and a parallel system is being set up in which the potential of the S3 approach and the S3 ecosystems has been overlooked. The interviewees speak of poor or non-existent multi-level governance, raising of unrealistic expectations, lack of alignment with regional needs, lack of consideration of existing territorial structures for strategic planning, and contradictory and confused information about the recovery plan drafting process. If we consider that without optimising the entrepreneurial ecosystem, industrial specialisation alone may not be successful (Szerb et al., 2020), we could agree that the opportunity to take advantage of the S3 quadruple helix ecosystem potential to contribute to a place-based industrial and societal recovery has probably been missed. And this may apparently be occurring not only in Spain but also in countries such as Italy or Croatia, as the European Committee of the Regions has warned on several occasions.^3^

When we asked the regional S3 practitioners what could have been done to give S3 a higher profile in the recovery process, they agreed that some more precise EC guidelines would probably have helped to secure the ex-ante connexion between recovery projects and S3 priorities. The truth is that the two funding schemes seem to have overlapped. If the challenge now, as Larosse et al. (2020) believe, is to recouple Smart Specialisation to a truly European transformation objective, serious consideration should have been given to the option of using Smart Specialisation strategies as an instrument to drive the transformative investments needed in an EU growth model defined by the digital and green transitions.

### The Need to Align Ex-post Recovery-S3 Priorities

During the conversations in the focus group meeting held 7 months after the interviews were conducted, all the regional S3 units confirmed that 6 months after Spain's Recovery and Resilience Plan (RRP) had been approved (30/04/2021); and 3 months after the Cohesion Regulations 2021–2027 had been published, no coordination or communication had been put in place between S3 regional practitioners and RRP managers. The origin of this mismatch was probably the complexity and immaturity of the RRP procedures and the rigidity of their timing. The truth is that, there is currently no alignment between the Spanish RRP and 2021–2027 Smart Specialisation strategies in the majority of the Spanish regions. This lack of communication at vertical National Government/Region level, but also at the horizontal level between the various regional departments in charge of both planning and implementation, has reinforced the feeling of isolation and may jeopardise what Grillitsch (2016) calls the “institutional harmony” which is necessary to build trust among institutions.

The following key contributions emerged as a result of the discussion in the focus group:

- Taking account of the qualified professional profile, skills and experience that regional S3 practitioners have in relation to innovative public policy approaches, such as the multi-actor and multi-level dimensions of S3, the lack of consideration of their added-value in the process of drafting the national recovery plan is viewed as a loss for an effective recovery process.
- As a consequence of the above point, the credibility and creditworthiness of regional S3 practitioners (and of the S3 approach itself) in the eyes of the regional S3 ecosystem is likely to be impaired.
- An opportunity has been also missed to ensure coherence between two multi-scalar planning processes (S3 working on the identification of regional priorities and the national recovery plan working on the identification of strategic projects in the country).
- The recovery process may leave behind some disadvantaged regions because their institutions have less capacity to contribute to the national calls launched under the Recovery Plan.
- Although the interviewees agree that coherence will eventually emerge in the implementation phase, participants felt a certain resignation at this situation. Even though they do expect an eventual ex-post alignment between the two processes, it will happen only if regional practitioners devote a considerable amount of time and resources to it, and they will have to deal with duplications and gaps in a very complex and time-constrained context (potentially resulting in less efficient public spending).

## Conclusions

On the basis of our results, there appears to be a need to bridge the gap caused by the S3 regional units and S3 ecosystem being left out of the response to the post-Covid recovery. In that connexion, we believe that S3 has to “reposition itself” in the logic of public intervention, especially at a time when there is a certain degree of disaffection with S3 processes on account of the lack of coherence with the recovery processes and the failure to include S3 regional ecosystems in the definition of those processes.

If we consider that once the EU makes a certain approach or policy compulsory, it must therefore be implemented in all Member States, it seems that an opportunity has been missed to make a strong statement about the role of S3 role in the current EU process; and with that, an opportunity for the potential of S3 to be consolidated as a contribution to the recovery process.

Much can be done, though, to ensure coherence between S3 and recovery projects, even a posteriori, by considering how the two planning processes complement one another, even if the challenge in relation to the debate over resilience vs. transformation has yet to be addressed.

Our results reveal a certain degree of resignation among regional S3 practitioners, but also a common belief in the possibilities for an ex-post alignment between the recovery strategies and the S3 priorities, allowing the collectively-built regional common visions to be protected. However, without a clear demarcation of responsibilities and sufficient political support to the S3 managing bodies, and without a clear recognition of the value created by regional actors belonging to the established S3 innovation ecosystems, the role that Smart Specialisation can play in the recovery process might be at risk. In that context, the S3 managing bodies need to be given more support and a stronger mandate to ensure effective multi-level governance and full consideration of place-based, multi-actor and inclusive approaches that exist in the regions.

Finally, in view of the strategic role that Smart Specialisation should play in the coming years, more serious consideration should be given to the opportunity to extend the S3 approach to all regional policies, beyond research and development. This would probably need further research to assess its potential impact on the framework of the EU green and digital transitions and on the European pillar of social rights, and also on the consolidation of a new role for European regional public actors and European regional innovation ecosystems as key facilitators in the recovery process.

## Data Availability Statement

The raw data supporting the conclusions of this article will be made available by the authors, without undue reservation.

## Author Contributions

All authors listed have made a substantial, direct, and intellectual contribution to the work and approved it for publication.

## Funding

This research has been supported by the University of Córdoba.

## Conflict of Interest

The authors declare that the research was conducted in the absence of any commercial or financial relationships that could be construed as a potential conflict of interest.

## Publisher's Note

All claims expressed in this article are solely those of the authors and do not necessarily represent those of their affiliated organizations, or those of the publisher, the editors and the reviewers. Any product that may be evaluated in this article, or claim that may be made by its manufacturer, is not guaranteed or endorsed by the publisher.

## Footnotes

^1^REGULATION (EU) 2021/1060 OF THE EUROPEAN PARLIAMENT AND OF THE COUNCIL of 24 June 2021 laying down common provisions on the European Regional Development Fund, the European Social Fund Plus, the Cohesion Fund, the Just Transition Fund and the European Maritime, Fisheries and Aquaculture Fund and financial rules for those and for the Asylum, Migration and Integration Fund, the Internal Security Fund and the Instrument for Financial Support for Border Management and Visa Policy.

^2^Committee of the Regions press release: https://cor.europa.eu/en/news/Pages/ECON-29-SEPTEMBRE2021.aspx.

^3^Press release: https://cor.europa.eu/en/news/Pages/ECON-29-SEPTEMBRE2021.aspx.
